# Essential role of the Na^+^-Ca2^+^ exchanger (NCX) in glutamate-enhanced cell survival in cardiac cells exposed to hypoxia/reoxygenation

**DOI:** 10.1038/s41598-017-13478-x

**Published:** 2017-10-12

**Authors:** Marta Maiolino, Pasqualina Castaldo, Vincenzo Lariccia, Silvia Piccirillo, Salvatore Amoroso, Simona Magi

**Affiliations:** Department of Biomedical Sciences and Public Health, School of Medicine, University “Politecnica delle Marche”, Via Tronto 10/A, 60126 Ancona, Italy

## Abstract

Myocardial ischemia culminates in ATP production impairment, ionic derangement and cell death. The provision of metabolic substrates during reperfusion significantly increases heart tolerance to ischemia by improving mitochondrial performance. Under normoxia, glutamate contributes to myocardial energy balance as substrate for anaplerotic reactions, and we demonstrated that the Na^+^/Ca^2+^ exchanger1 (NCX1) provides functional support for both glutamate uptake and use for ATP synthesis. Here we investigated the role of NCX1 in the potential of glutamate to improve energy metabolism and survival of cardiac cells subjected to hypoxia/reoxygenation (H/R). Specifically, in H9c2-NCX1 myoblasts, ATP levels, mitochondrial activities and cell survival were significantly compromised after H/R challenge. Glutamate supplementation at the onset of the reoxygenation phase significantly promoted viability, improved mitochondrial functions and normalized the H/R-induced increase of NCX1 reverse-mode activity. The benefits of glutamate were strikingly lost in H9c2-WT (lacking NCX1 expression), or in H9c2-NCX1 and rat cardiomyocytes treated with either NCX or Excitatory Amino Acid Transporters (EAATs) blockers, suggesting that a functional interplay between these transporters is critically required for glutamate-induced protection. Collectively, these results revealed for the first time the key role of NCX1 for the beneficial effects of glutamate against H/R-induced cell injury.

## Introduction

Myocardial ischemia refers to a restriction in blood flow to the heart causing a shortage of oxygen and substrates supply, which in turn affects mitochondrial respiratory chain, aerobic metabolism and, consequently ATP production. Although the prompt restoration of blood flow salvages myocardium that would otherwise succumb to necrosis, reperfusion imposes its own set of injury-promoting challenges, known as “reperfusion injury”^1,2^. Over the last years, different approaches have been explored to minimize further infarct size progression and thereby improve outcomes in the aftermath of myocardial ischemia/reperfusion (I/R)^3^. In particular, interventions during the reperfusion are feasible strategies for cardioprotection, and the resumption of the aerobic metabolism through the provision of energy substrates is one of the most promising approach^4^. In this regard, experimental and clinical evidence suggest that glutamate supplementation has the potential to protect myocardium against I/R injury^5–7^. Glutamate is a key molecule in cellular metabolism^8,9^: it can fuel respiration and participate as anaplerotic substrate to maintain optimum levels of Krebs cycle intermediates, which are typically compromised in the ischemic heart^10,11^, or even provide cellular energy through substrate level phosphorylation reactions^4^. A decrease in glutamate myocardial concentrations has been observed during and after ischemic insults both in animals and human studies^12,13^, as a possible consequence of its enhanced metabolic utilization^14,15^ or exacerbated leak from myocytes^16^. However, a clear understanding of the molecular machinery involved in metabolic responses activated by glutamate in ischemic settings is still lacking.

We have recently demonstrated that in physiological conditions glutamate supplementation increases ATP cellular content through a mechanism that involves both the Na^+^/Ca^2+^ exchanger (NCX) and the Na^+^ dependent Excitatory Amino Acid Transporters (EAATs), in neuronal, glial and cardiac models^17,18^. Specifically, we reported a functional interaction between NCX1 and the Excitatory Amino Acid Carrier 1 (EAAC1), both at plasma membrane and mitochondrial level, where these transporters cooperate in order to favor glutamate entry into the cytoplasm and then into the mitochondria, thereby enhancing ATP synthesis^17,18^. Based on these findings, we explored the hypothesis that glutamate supplementation during the reoxygenation phase improves the recovery of metabolic activity and cell survival in cardiac cells subjected to hypoxia/reoxygenation (H/R), and that NCX1 coupling to EAATs is critically involved.

## Results

### Effect of glutamate on H/R injury: involvement of NCX1

**Figure 1:**
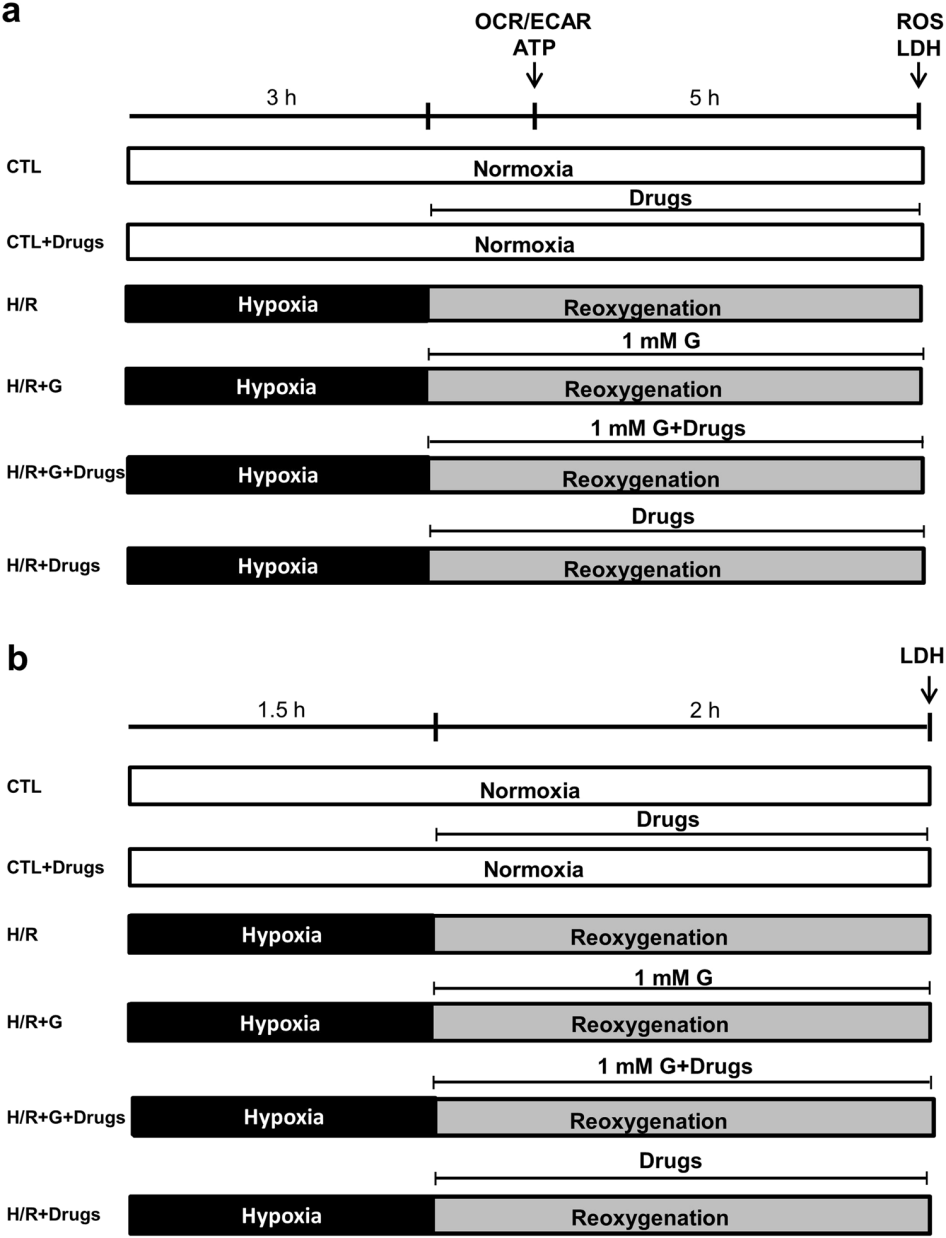
Timeline of the experimental protocols (H/R). Schematic diagram showing the H/R timeline protocol in H9c2 cells (**a**) and in isolated rat adult cardiomyocytes (**b**). Control groups were incubated under normoxic conditions at 37 °C for the entire protocol. Glutamate (1 mM)-alone or in combination with 1 µM SN-6, 300 µM DL-TBOA or 3 μg/ml oligomycin (for ATP experiments conducted in H9c2-NCX1 cells)-was administered during the reoxygenation phase. Cell viability (assessed by extracellular LDH measurement) and ROS were evaluated at the end of the reoxygenation phase in both experimental protocols. ATP content, OCR and ECAR were assessed after the first hour of reoxygenation in H9c2-NCX1 cells. CTL = control; H/R = hypoxia/reoxygenation; G = glutamate.

**Figure 2:**
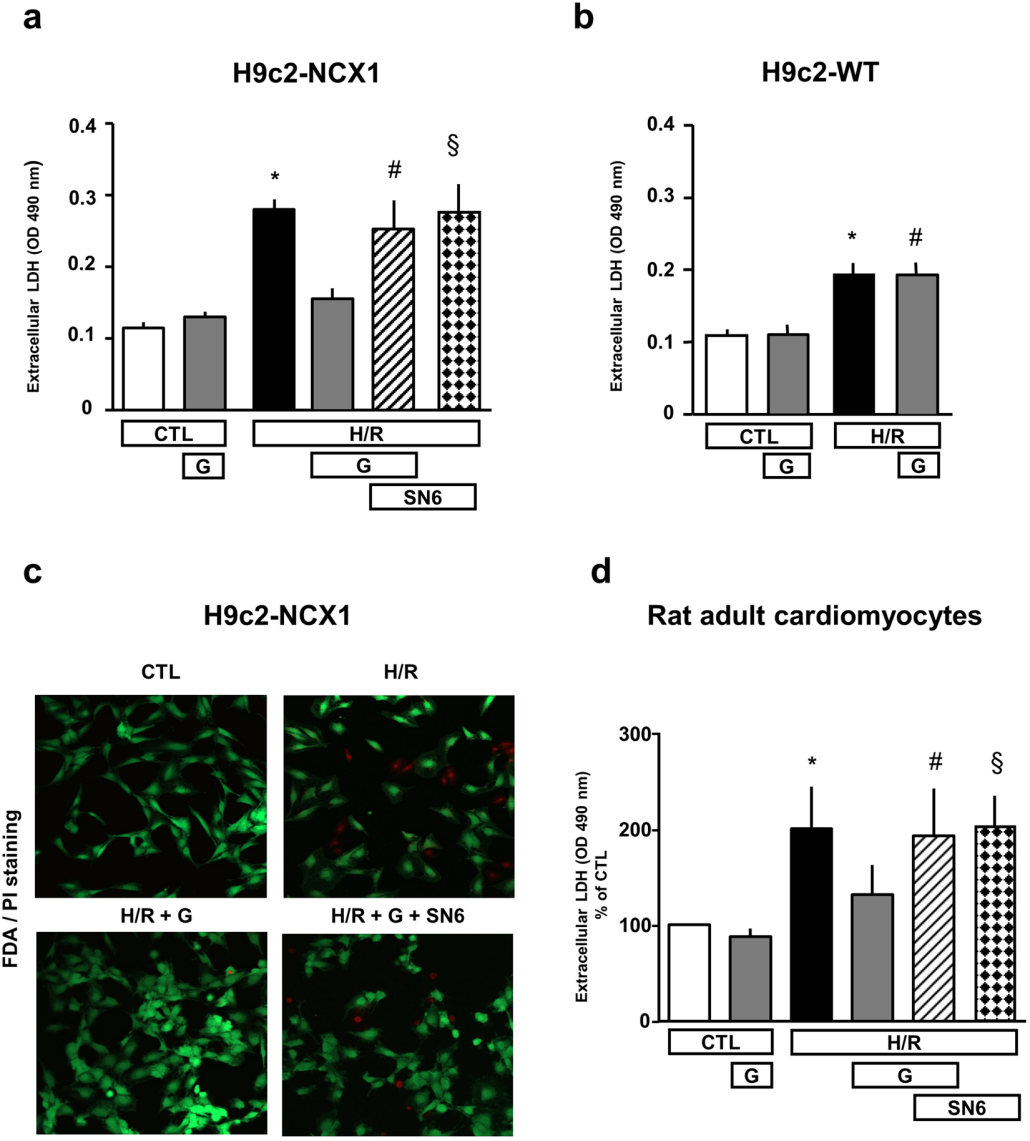
Effect of NCX inhibition on glutamate-induced protection against H/R injury. Extracellular LDH activity measured 5 h after the hypoxic insult (3 h) in H9c2 cells (**a**,**b**) and 2 h after the hypoxic insult (1.5 h) in rat adult cardiomyocytes (**d**) in different experimental conditions. 1 mM glutamate, alone or in combination with 1 μM SN-6, was added during the reoxygenation phase. Differences among means were assessed by one-way ANOVA followed by Dunnet’s *post hoc* test. Each column represents the mean ± S.E.M. of almost 5 independent experiments performed in duplicate. (**a**) *p < 0.001 versus CTL, CTL + G and H/R + G; ^#^p < 0.001 versus CTL, p < 0.01 versus CTL + G and p < 0.05 versus H/R + G; ^§^p < 0.001 versus CTL and CTL + G, p < 0.01 versus H/R + G. (**b**) *p < 0.01 versus CTL and CTL + G; ^#^p < 0.001 versus CTL and p < 0.01 versus CTL + G. (**d**) LDH levels were normalized to the control (normoxia-exposed) group and expressed as percentage. Each column represents the mean ± S.E.M. of almost 4 independent experiments performed in duplicate. *p < 0.001 versus CTL and CTL + G and p < 0.05 versus H/R + G; ^#^p < 0.001 versus CTL + G, p < 0.01 versus CTL and p < 0.05 versus H/R + G; ^§^p < 0.001 versus CTL and CTL + G, and p < 0.05 versus H/R and H/R + G. (**c**) Analysis of H9c2 cell survival by FDA/PI staining. Images are representative of 3 independent experiments. CTL = control; H/R = hypoxia/reoxygenation; G = glutamate.

We initially established an *in vitro* model of H/R based on two H9c2 clones^19^, H9c2-WT (not expressing endogenous NCX1 under our culture conditions^17,20^ and H9c2-NCX1 (generated from H9c2-WT and stably expressing canine NCX1^17^). When cells were subjected to 3 h of hypoxia followed by 5 h of reoxygenation (Fig. 1a), we found that cell damage, as assessed by extracellular LDH levels^19^ and fluorescein diacetate/propidium iodide (FDA/PI) double staining^21,22^, was significantly higher in both H9c2 cell lines than their respective normoxic controls (Fig. 2a,b and Supplementary Fig. 1). To study whether glutamate attenuates H/R injury and assess the specific contribution of NCX1, H9c2 cells were treated with glutamate at the onset of the reoxygenation phase. Although H9c2-NCX1 cells are even more vulnerable to H/R than H9c2-WT (Fig. 2a,b and Supplementary Fig. 1), as previously reported^19^, glutamate supplementation during the reoxygenation phase fully prevented H/R damage only in H9c2-NCX1 but not in H9c2-WT cells (Fig. 2a,b). Notably, glutamate at the concentration used (1 mM) was devoid of detectable toxicity under normoxic conditions (Fig. 2). Further evidence that a functional NCX1 is determinant for glutamate protection was obtained by evaluating the efficacy of glutamate to limit H/R injury after pharmacological blockade of NCX1. In particular, when H9c2-NCX1 cells were exposed to the selective NCX inhibitor 2-[[4-[(4Nitrophenyl) methoxy] phenyl] methyl]-4-thiazolidinecarboxylic acid ethyl ester (SN-6)^23,24^ (1 µM) during the reoxygenation phase, glutamate was wholly ineffective in protecting cells against H/R injury (Fig. 2a,c). SN-6 *per se* has no effect on H9c2-NCX1 cell viability under normoxia^19^ or when introduced only at the reperfusion during our H/R protocol (Figs 1 and 2a,c). Noteworthy, the same results were obtained in primary culture of rat adult cardiomyocytes, which endogenously express NCX1. When cardiomyocytes were subjected to the H/R protocol^19^ shown in Fig. 1b, we found that 1 mM glutamate greatly reduced H/R-induced cell damage, and that glutamate-induced protection was lost in the presence of SN-6 (Fig. 2d). In line with results obtained in H9c2-NCX1 cells, SN-6 during the reoxygenation phase had no effect on cell survival, as well as cardiomyocytes viability was not compromised by glutamate when used at 1 mM concentration under normoxic conditions (Fig. 2d).

### Effect of glutamate on H/R injury: involvement of EAATs

**Figure 3:**
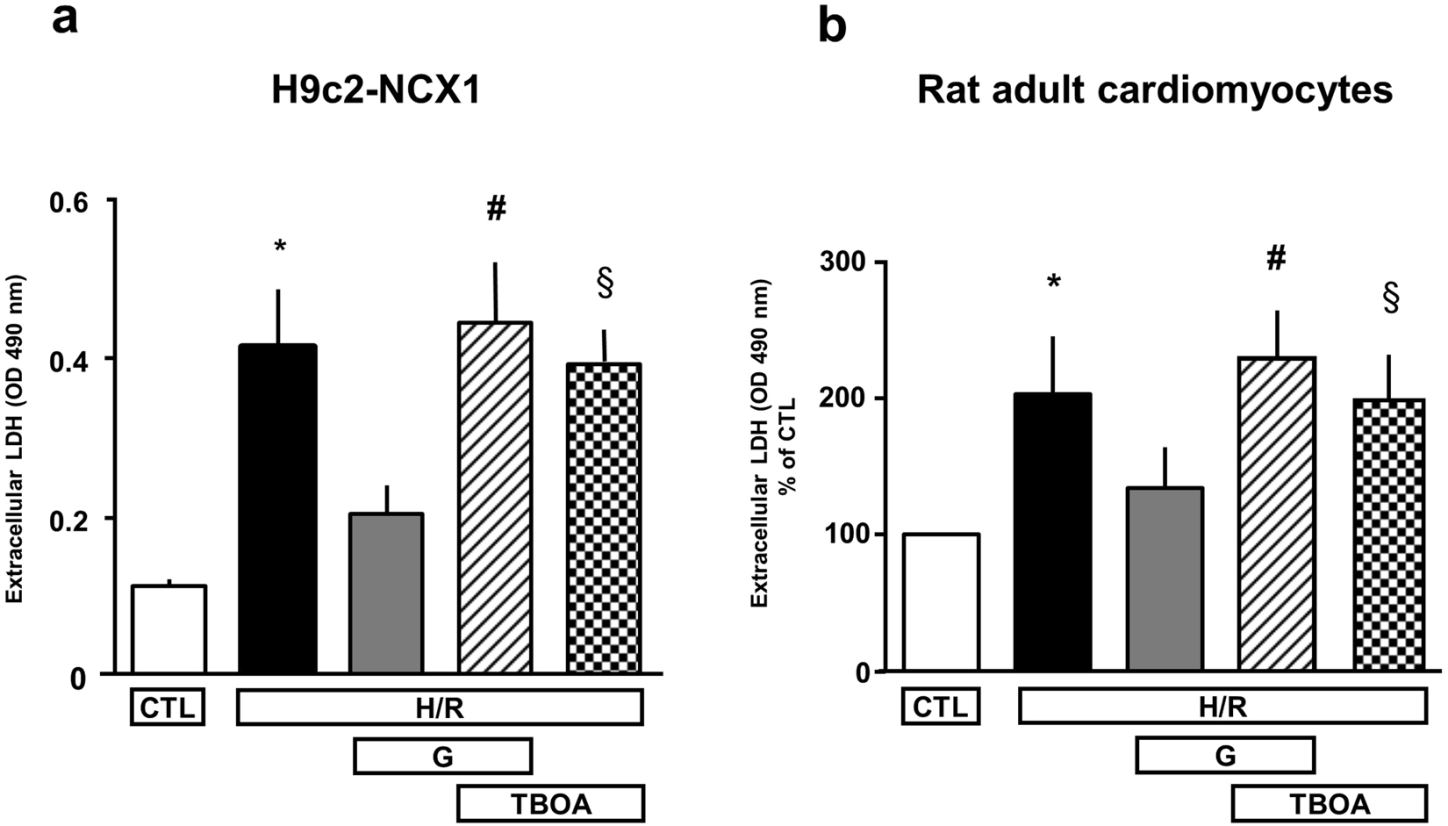
Effect of EAATs inhibition on glutamate-induced protection against H/R injury. Extracellular LDH activity measured 5 h after the hypoxic insult (3 h) in H9c2-NCX1 cells (**a**) and 2 h after the hypoxic insult (1.5 h) in rat adult cardiomyocytes (**b**) in different experimental conditions. 1 mM glutamate, alone or in combination with 300 μM DL-TBOA, was added during the reoxygenation phase. Differences among means were assessed by one-way ANOVA followed by Dunnet’s *post hoc* test. (**a**) Each column represents the mean ± S.E.M. of almost 6 independent experiments performed in triplicate. *p < 0.001 versus CTL and p < 0.01 versus H/R + G; ^#^p < 0.001 versus CTL and p < 0.01 versus H/R + G; ^§^p < 0.001 versus CTL and p < 0.01 versus H/R + G. (**b**) LDH levels were normalized to the control (normoxia-exposed) group and expressed as percentage. Each column represents the mean ± S.E.M. of 4 independent experiments performed in duplicate. *p < 0.001 versus control groups and p < 0.05 versus H/R + G; ^#^p < 0.001 versus control groups and H/R + G; ^§^p < 0.01 versus CTL and p < 0.05 versus H/R + G. CTL = control; H/R = hypoxia/reoxygenation; G = glutamate; TBOA = DL-TBOA.

It is widely accepted that plasma membrane Excitatory Amino Acid Transporters (EAATs) are primarily responsible for glutamate entry into the cells^25^. We have recently demonstrated that, in particular physiological conditions, glutamate entry into the cells through EAATs relies upon NCX activity^17^. Thus, once we have demonstrated that a functional NCX1 was required for glutamate-induced protection against H/R injury, we investigated EAATs involvement in this phenomenon. To this aim, we used the non-transportable EAATs blocker DL-*threo*-β-Benzyloxyaspartic acid (DL-TBOA)^26,27^ at the concentration of 300 µM. Exposure to glutamate for the entire reoxygenation phase failed to prevent H/R-induced cell death in the presence of DL-TBOA (Fig. 3a). Same results were obtained in primary culture of rat adult cardiomyocytes (Fig. 3b). These findings support the substantial role of EAATs in the observed glutamate-induced protective response. DL-TBOA *per se* does not affect cell viability neither in normoxia (data not shown) nor after H/R (Fig. 3).

### Effect of glutamate exposure on ATP production

**Figure 4:**
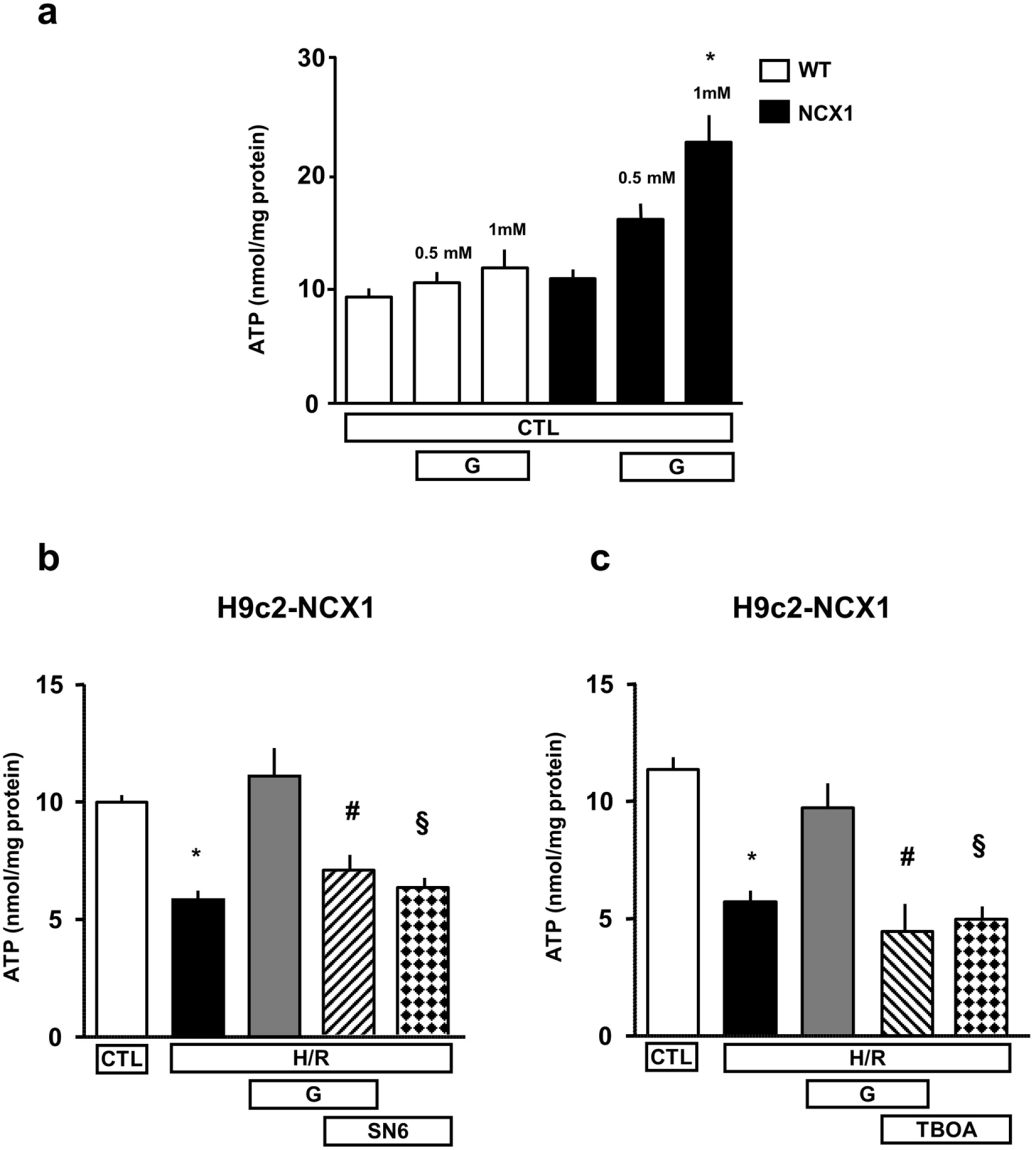
Effect of NCX and EAATs inhibition on glutamate-induced ATP synthesis in H9c2-NCX1 cells subjected to H/R. (**a**) Intracellular ATP levels evaluated under normoxic conditions in both H9c2-WT and H9c2-NCX1 cells exposed to different glutamate concentrations (0.5 and 1 mM) for 1 h. (**b**,**c**) Intracellular ATP levels evaluated in different experimental conditions. 1 mM glutamate, alone or in combination with 1 μM SN-6 or 300 µM DL-TBOA, was added during the reoxygenation phase, then ATP levels were monitored after 1 h. ATP levels were normalized to the respective sample protein content. Differences among means were assessed by one-way ANOVA followed by Dunnet’s *post hoc* test. Each column represents the mean ± S.E.M. of almost 10 independent experiments performed in triplicate. (**a**) *p < 0.001 versus CTL and p < 0.05 versus 0.5 mM. (**b**) *p < 0.001 versus CTL and H/R + G; ^#^p < 0.05 versus CTL and p < 0.001 versus H/R + G; ^§^p < 0.01 versus CTL and p < 0.001 versus H/ + G. (**c**) *p < 0.001 versus CTL and H/R + G; ^#^p < 0.001 versus CTL and H/R + G; ^§^p < 0.001 versus CTL and H/R + G. CTL = control; H/R = hypoxia/reoxygenation; G = glutamate; TBOA = DL-TBOA.

Thereafter, we explored the potential mechanism underlying the protective action exerted by glutamate and the role of NCX1. We recently reported that, in physiological conditions, NCX activity supports glutamate-enhanced ATP synthesis in several cell models^17,18^. Therefore, we investigated whether this effect could be involved in the recovery of cardiac metabolism from hypoxic state. We tested this hypothesis in our experimental model, first in normoxia and then during H/R. As shown in Fig. 4a, when cells were exposed to different glutamate concentration (0.5 and 1 mM) for 1 h, a remarkable increase in ATP synthesis occurred in H9c2-NCX1 (nmol/mg protein: 15.9 ± 0.61 and 22.6 ± 0.97 versus 10.8 ± 0.26, for glutamate 0.5 and 1 mM, respectively) but not in H9c2-WT cells (nmol/mg protein: 10.5 ± 0.34 and 11.8 ± 0.58 versus 9.2 ± 0.28, for glutamate 0.5 and 1 mM, respectively), in line with our previous results^17^. Given that H9c2-WT cells were refractory to glutamate stimulation (Fig. 2b and Fig. 4a), we further analyzed H9c2-NCX1 cells. We tested the ability of glutamate to fuel ATP recovery during reoxygenation after hypoxia, and explored whether a NCX/EAAT functional coupling could play any role. For this set of experiments, we used glutamate at 1 mM according to the protocols described in Fig. 1. As shown in Fig. 4b, in cells underwent to H/R injury, the ATP content was significantly reduced compared to the control (nmol/mg protein: 5.8 ± 0.3 versus 10.0 ± 0.3). Glutamate administration during the first hour of reoxygenation evoked a raise in ATP production up to the levels observed under normoxic conditions (nmol/mg protein: 11.0 ± 1.1 versus 10.0 ± 0.3). Interestingly, the ability of glutamate to restore ATP levels was abolished by SN-6 (nmol/mg protein: 11.0 ± 1.1 versus 6.84 ± 0.66), suggesting that NCX1 activity is critical for the metabolism recovery promoted by glutamate during the reoxygenation. Since we observed that the protective effect of glutamate relied both on NCX1 and EAATs (Figs 2 and 3), which functionally interact to allow glutamate entry into the cytosol, improving the energetic balance of the cells^17,18^, we also tested whether the ATP response to glutamate during the reoxygenation was prevented by DL-TBOA (300 µM). The analysis of the cellular ATP content revealed that the ability of glutamate to restore ATP levels after hypoxia was abolished by DL-TBOA addition (Fig. 4c) confirming the involvement of EAATs in such glutamate-induced protective response. Both SN-6 and DL-TBOA do not affect ATP levels neither in normoxia^17^ nor after H/R protocol (Fig. 4b,c).

### Effect of glutamate on mitochondrial function following H/R challenge

**Figure 5:**
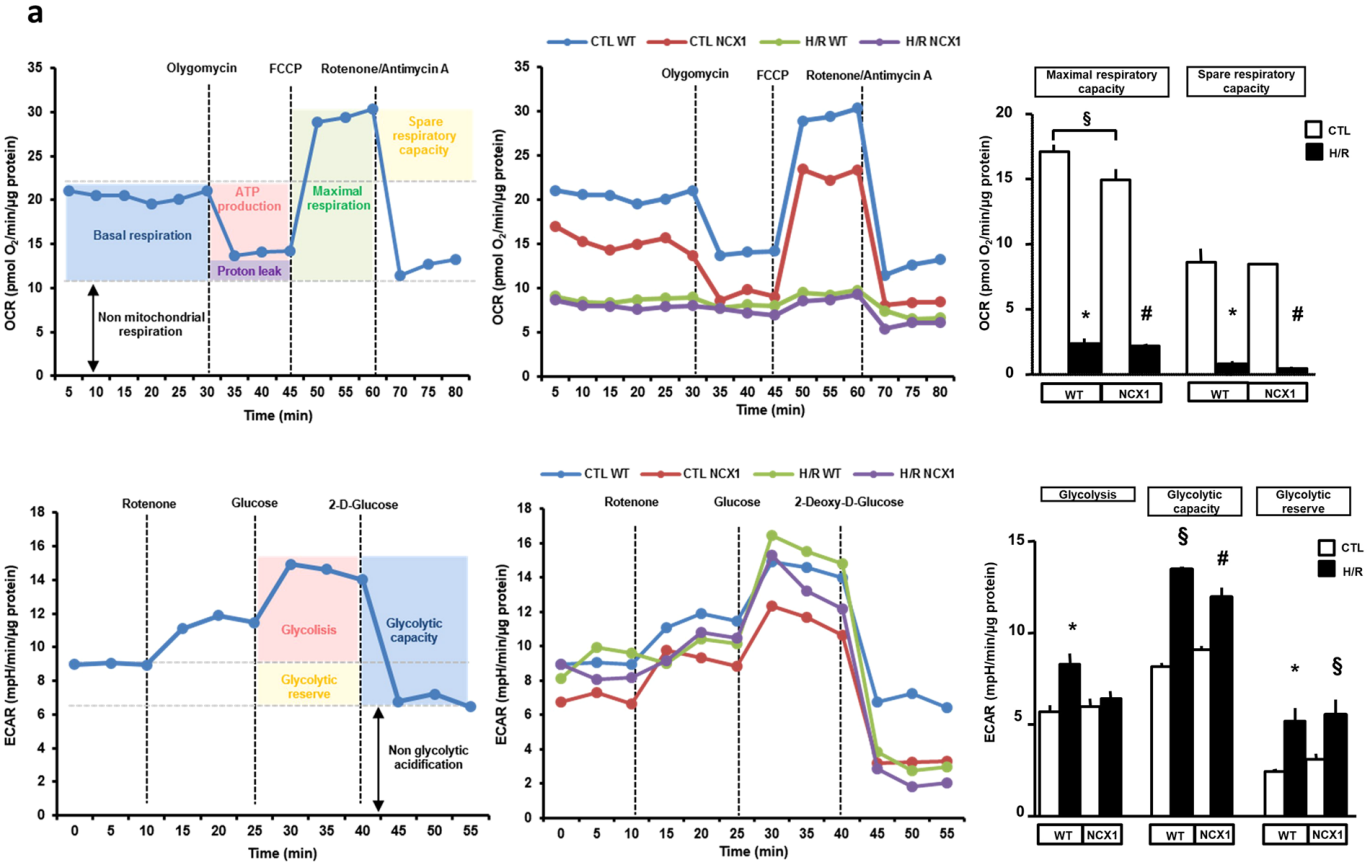
Energy metabolism characterization in H9c2-WT and H9c2-NCX1 cells. Oxidative phosphorylation rate (**a**) and glycolysis rate (**b**) determined by Seahorse XF24 Extracellular Flux Analyzer in H9c2-WT and H9c2-NCX1 cells exposed to 3 h of hypoxia followed by 1 h of reoxygenation. Each column represents the mean ± S.E.M. of 3 replications. Where unseen, error bars overlap with the histogram outline. Differences among means were assessed by Student’s *t-test*. Left panels show representative traces of both OCR and ECAR experiments. Representative profiles of the Seahorse assays are also shown. (**a**) Maximal respiratory capacity: ^§^P < 0.05; *p < 0.001 and ^#^p < 0.001 versus the respective control. Spare respiratory capacity: *p < 0.001 and ^#^p < 0.001 versus the respective control. (**b**) Glycolysis: *p < 0.01 versus the respective control. Glycolytic capacity: ^§^p < 0.001 and p < 0.001 versus the respective control. Glycolytic reserve: *p < 0.01 and ^§^p < 0.01 versus the respective control. CTL = control; H/R = hypoxia/reoxygenation.

**Figure 6:**
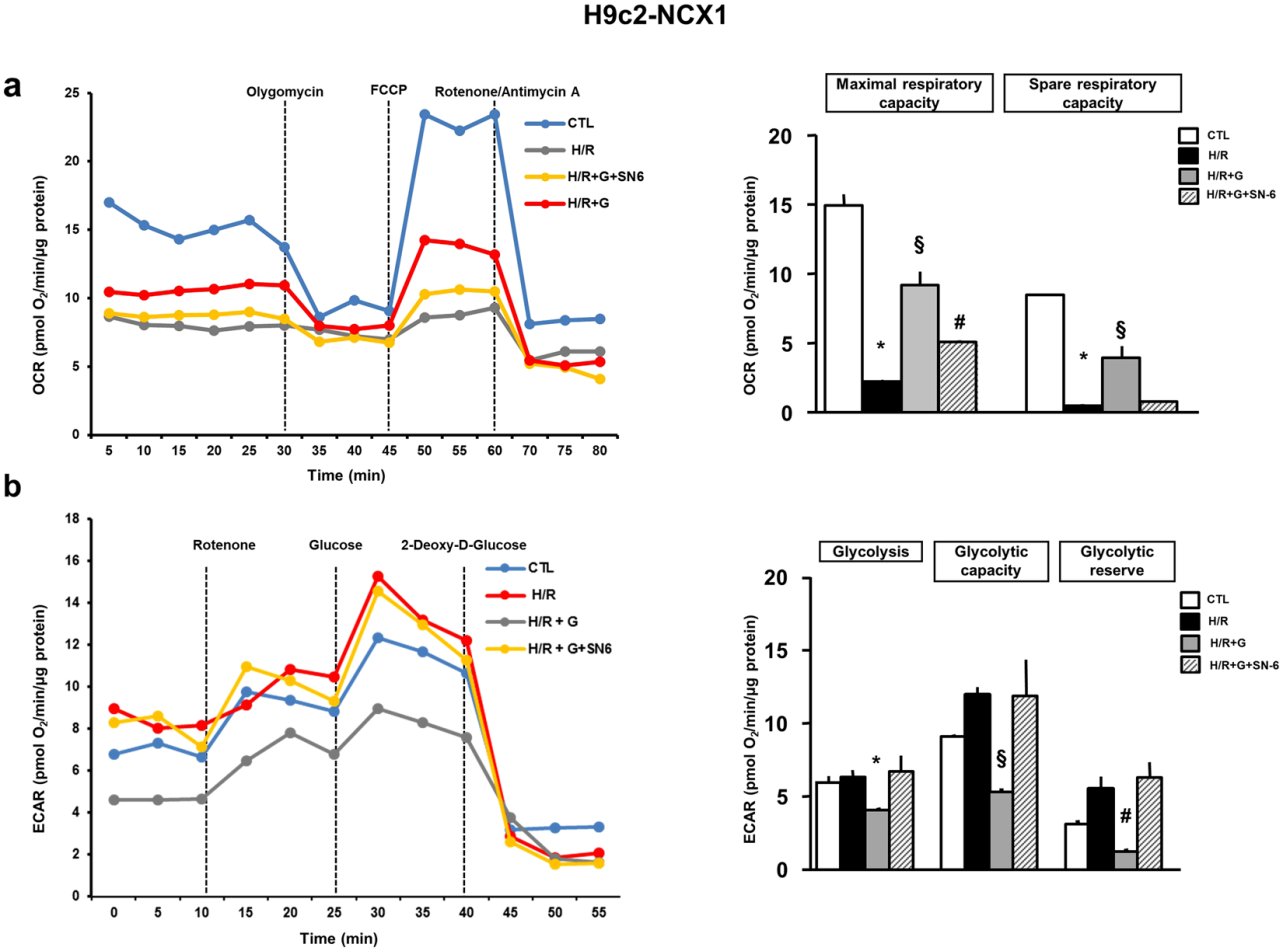
Effect of glutamate supplementation and NCX1 inhibition on cellular metabolism in H9c2-NCX1 cells subjected to H/R challenge. Oxidative phosphorylation rate (**a**) and glycolysis rate (**b**) determined by Seahorse XF24 Extracellular Flux Analyzer in H9c2-NCX1 cells exposed to 3 h of hypoxia followed by 1 h of reoxygenation in different experimental conditions. 1 mM glutamate, alone or in combination with 1 μM SN-6, was added during the reoxygenation phase and maintained for 1 h. Each column represents the mean ± S.E.M. of 4 replications. Where unseen, error bars overlap with the histogram outline. Differences among means were assessed by one-way ANOVA followed by Dunnet’s *post hoc test*. Left panels show representative traces of both OCR and ECAR experiments. (**a**) Maximal respiratory capacity: *p < 0.001 versus CTL and H/R + G, p < 0.01 versus H/R + G + SN-6; ^§^p < 0.001 versus all groups; ^#^p < 0.001 versus CTL and H/R + G, p < 0.01 versus H/R. Spare respiratory capacity: *p < 0.001 versus CTL and H/R + G; ^§^p < 0.001 versus all groups. (**b**) Glycolysis: *p < 0.05 versus CTL, p < 0.01 versus H/R and H/R + g + SN-6. Glycolyitic capacity: ^§^p < 0.05 versus CTL, p < 0.001 versus H/R and H/R + G + SN-6. Glycolytic reserve: *p < 0.01 versus CTL; ^#^p < 0.05 versus CTL, p < 0.001 versus H/R and H/R + G + SN-6. CTL = control; H/R = hypoxia/reoxygenation.

The set of experiments presented here were performed in order to further (a) characterize the metabolic response to glutamate in the post-hypoxic phase, (b) assess whether glutamate-induced protection could be related to an improvement of oxidative metabolism, (c) explore the role of NCX1 in mitochondrial responses to glutamate. We first probed the metabolic phenotype of H9c2-WT and H9c2-NCX1 cells by analyzing mitochondrial respiration, assessed as oxygen consumption rate (OCR) and glycolytic activity, measured as extracellular acidification rate (ECAR) of the surrounding media (which predominately reflects the excretion of lactic acid converted from pyruvate). Experimental operative protocols are summarized in Fig. 5 (see “Methods” for further details). In normoxic conditions, ECAR at baseline was not different between H9c2-WT and H9c2-NCX1 cells, whereas maximal respiratory capacity was slightly but significantly smaller in H9c2-NCX1 cells (Fig. 5a). When H/R stress was applied, both cell lines showed a marked decrease in mitochondrial oxygen consumption that was better compensated in H9c2-WT by an increase in glycolysis (Fig. 5b). Next, we measured OCR and ECAR parameters in hypoxic H9c2-NCX1 cells reoxygenated with glutamate. As shown in Fig. 6a, after glutamate treatment, OCR profiles greatly improved toward normoxic values. In particular, in H9c2-NCX1 cells reoxygenated in the presence of glutamate we observed a significant recovery of maximal respiratory capacity, which indicates increased activity of the electron transport chain (ETC), as well as a significant improvement in spare respiratory capacity, which estimates cell’s ability to cope with large increases in energy demand and reflects the amount of extra ATP that can be produced by oxidative phosphorylation. Notably, the recovery of OCR profiles induced by glutamate was significantly inhibited when NCX1 was blocked with SN-6 during the reoxygenation phase (Fig. 6a). Glutamate ability to promote mitochondrial ATP generation (and thereby survival) in H/R H9c2-NCX1 cells was also supported by the capacity of oligomycin^18^ (an inhibitor of the ATP synthase, the final enzyme in the oxidative phosphorylation pathway) to fully prevent the ATP response to glutamate during reoxygenation phase (Supplementary Fig. 2). Finally, as shown in Fig. 6b, glycolytic activity was significantly reduced by glutamate during H/R in H9c2-NCX1 cells, and also this metabolic response was sensitive to NCX1 blockade. Collectively, these data lend further support to the hypothesis that NCX1 is critical for the glutamate-dependent metabolic boost in myocytes recovering from hypoxic insult, and that the ATP production stimulated by glutamate during the reoxygenation phase essentially relies on mitochondrial oxidative phosphorylation.

### Analysis of NCX 1 and EAATs expression following H/R challenge

**Figure 7:**
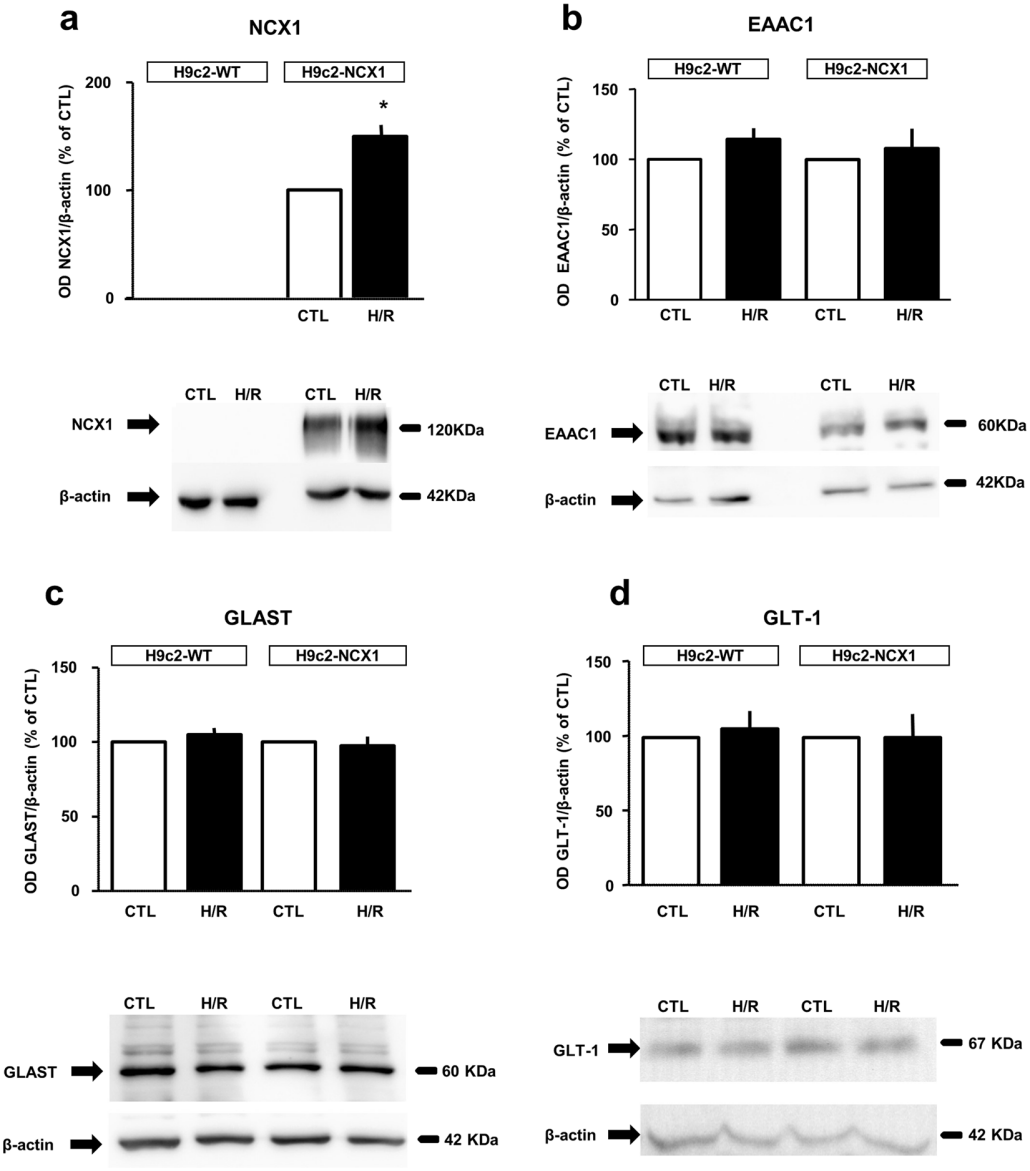
NCX1 and EAATs expression in H9c2-WT and H9c2-NCX1 cells subjected to H/R challenge. Quantitative densitometry showing the expression of NCX1 (**a**) and the Na^+^-dependent glutamate transporters EAAC1 (**b**), GLAST (**c**) and GLT-1 (**d**) in H9c2-WT and H9c2-NCX1 cells exposed to H/R. β-actin was used as loading control. Normalized optical density values are expressed as percentage of the respective control. Each column represents the mean ± S.E.M. of 3 independent experiments. Differences among means were assessed by Student’s *t-test*. (**a**) *p < 0.01 versus CTL. Representative western blot images are shown below. Full-length blots are presented in Supplementary Fig. S4. CTL = control; H/R = hypoxia/reoxygenation.

In our previous studies, we demonstrated that both in the heart and in H9c2-NCX1 cells NCX1 protein expression is upregulated under stressful conditions, including hypertrophy and ischemic injury^19,20^. Since we observed that the protective effect of glutamate against H/R damage relied both on NCX1 and EAAT activities (Figs 2 and 3), we explored whether EAATs expression could also be modified by H/R challenge. As shown in Fig. 7, protein expression analysis revealed that, in H9c2-NCX1 cells, NCX1 levels were increased after H/R (Fig. 7a), in line with our previous report^19^. Again, in H9c2-WT cells NCX1 protein expression was undetectable both in normoxia and after H/R challenge (Fig. 7a). The expression of the three main EAATs expressed in H9c2 cells^17^, namely EAAC1, GLAST and GLT-1 was unmodified after H/R challenge, in both H9c2-WT and H9c2-NCX1 cells (Fig. 7b–c).

### Effect of glutamate on NCX1 activity alteration following H/R

**Figure 8:**
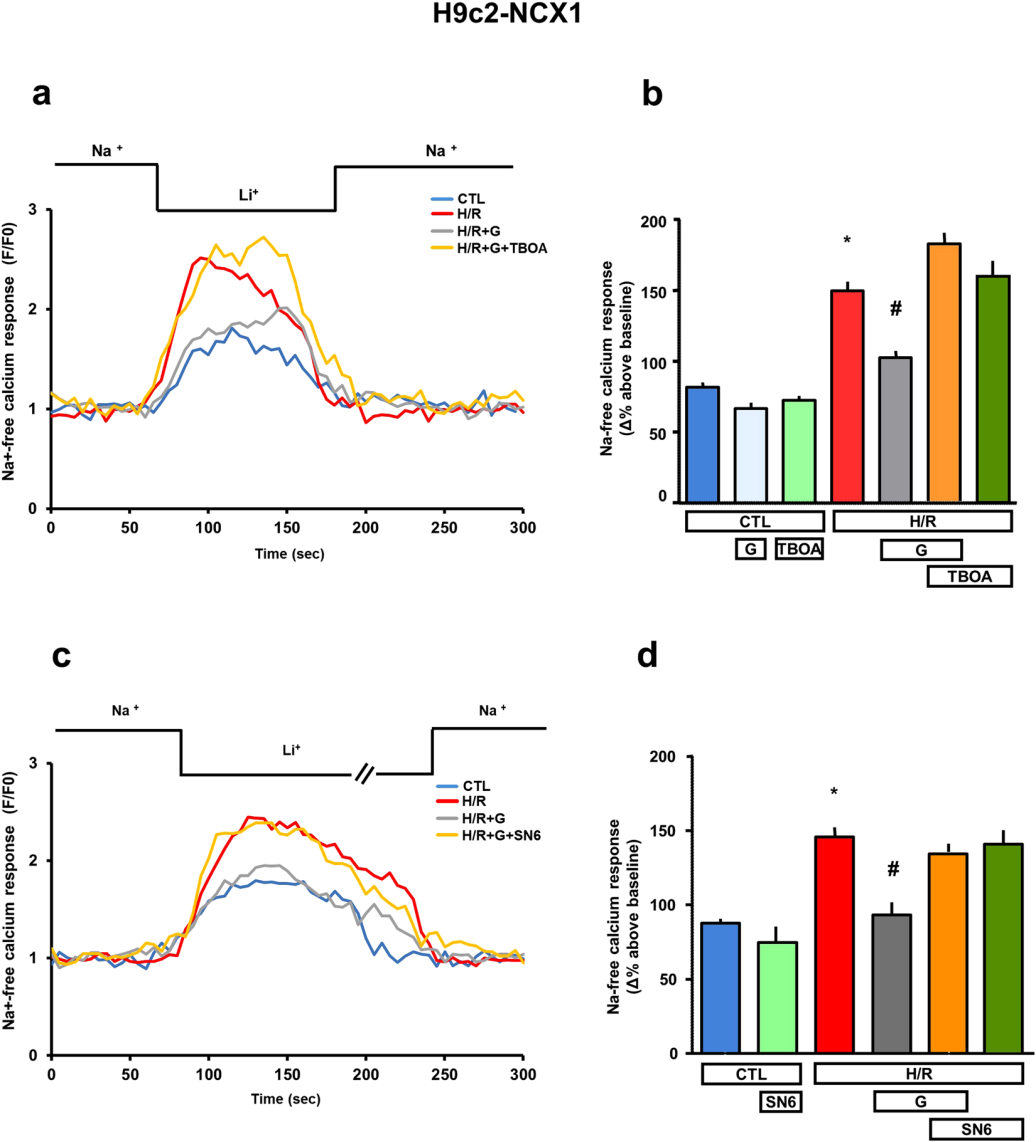
Effect of glutamate exposure on NCX activity in H9c2-NCX1 cells subjected to H/R. (**a**,**c**) Representative records of Ca^2+^ responses to Na^+^-free challenge after incubation in normoxic conditions (blu line), H/R insult (red line), and after H/R challenge in the presence of 1 mM glutamate during the entire reoxygenation phase alone (grey line) and in combination with the EAATs and NCX inhibitors (yellow line) DL-TBOA (300 µM) and SN-6 (1 µM), respectively. Fluorescence intensity was expressed as F/F0-ratio, where F is the background subtracted fluorescence intensity and F0 is the background subtracted mean fluorescence value measured from each cell at resting conditions (F/F0). (**b**,**d**) Analysis of the reverse mode of NCX1 activity in the presence of 1 mM glutamate, 300 µM DL-TBOA and 1 µM SN-6 in normoxic and hypoxic conditions. The NCX1 reverse mode activity was expressed as percentage of resting condition (∆%) after H/R protocol. For Δ% calculation, we used the maximal value of fluorescence obtained after stimulation and, as baseline, the mean of fluorescence recorded during the 30 seconds preceding the Na^+^-free challenge. Differences among means were assessed by one-way ANOVA followed by Dunnet’s *post hoc* test. The bar plot reports the mean ± S.E.M. of the [Ca^2+^]_i_ increase elicited by Na^+^-free pulse. For each experimental group, 100–200 cells were recorded in different experimental sessions. (**b**) *p < 0.001 versus all groups except versus H/R + TBOA and HR + G + TBOA (not significant); ^#^p < 0.01 versus all groups except versus all control groups. (**d**) *p < 0.001 versus all groups except versus H/R + SN-6 and H/R + G + SN-6 (not significant); ^#^p < 0.01 versus all groups except versus all control groups (not significant). CTL = control; H/R = hypoxia/reoxygenation; G = glutamate; TBOA = DL-TBOA.

Cardiac ischemia typically increases expression of NCX1^19^, and the accompanying alterations of exchange activity during I/R injury fuel a vicious circle of further damage by promoting intracellular Ca^2+^ overload^28,29^. We therefore verified that NCX1 function was altered in our H/R model and explored the possibility that the normalization of its activity accompanies the protection induced by glutamate. Exchanger activity was monitored in Fluo-4 loaded H9c2-NCX1 cells subjected to isotonic extracellular Na^+^ removal at the end of the experimental protocol reported in Fig. 1 (see “Methods” for further details). No change in fluorescence baseline was observed in H9c2-WT cells subjected to the same Na^+^ removal protocol (data not shown)^17^, confirming that the Ca^2+^ responses observed in H9c2-NCX1 are mediated by NCX1 reverse mode. As shown in Fig. 8, when NCX1 reverse mode was activated by superfusing a Na^+^-free extracellular solution, in control cells a rise in intracellular Ca^2+^ concentration ([Ca^2+^]_i_) of about 80% occurred, as revealed by the increase in fluorescence signal. When H/R group was analyzed, we observed that the NCX1-mediated increase in [Ca^2+^]_i_ was enhanced (about 50%) compared to what observed in control cells. When glutamate was added during the entire reoxygenation phase, NCX1 reverse activity was normalized to normoxic values and was significantly reduced compared to H/R group. Glutamate failed to prevent the H/R-induced increase of NCX1 reverse activity in the presence of both DL-TBOA and SN-6. As shown in Fig. 8b,d, the inhibitors do not affect *per se* Ca^2+^ responses, neither in normoxia nor in H/R conditions.

## Discussion

In this report we provide evidence that glutamate supplementation from the start of the reoxygenation phase counteracted the H/R-induced injury in cardiac cells. In particular, we demonstrated that such glutamate protective action was related to its ability to sustain oxidative metabolism, leading to an increase in ATP cellular content. Noteworthy, we showed that this protection disappeared in the absence of a functional NCX1, disclosing a key role of this transporter in sustaining cell viability.

NCX1 is central to many pathophysiological functions of the heart^19,20,30,31^, and in particular its role in cardiac ischemia has been investigated in different *in vitro* and *in vivo* models^30^. On one hand, a detrimental role of NCX1 *during* myocardial I/R emerges when the unbalanced exchange activity contributes to myocyte electrical instability and promotes Ca^2+^ overload^28,29^, so those strategies that ultimately normalize NCX1-mediated Na^+^ and Ca^2+^ ionic fluxes have therapeutic potential^32^. We have previously found in different cardiac models that pharmacological blockade of NCX1throughout the entire H/R protocol is protective^19^. On the other hand, NCX1 activity is strategic for cardioprotection against I/R evoked by conditioning programs^19,33^. Intriguingly, we disclosed here a new beneficial and essential role of NCX1 in glutamate-induced cell survival in cardiac models of H/R. This conclusion is supported by the following evidence: (1) in H9c2 cells, glutamate productively sustained mitochondrial ATP synthesis thereby promoting survival in H9c2-NCX1 (expressing NCX1) but not in H9c2-WT (not expressing NCX1)^17^ (Figs 2 and 4); (2) in H9c2-NCX1 cells, pharmacological blockade of NCX1 during the reoxygenation phase completely prevented the beneficial effects of glutamate in terms of recovery of ATP synthesis (Fig. 4) and mitochondrial respiration (Fig. 6), cell protection (Fig. 2) and normalization of Na^+^/Ca^2+^exchanger activity (Fig. 8); (3) in rat adult cardiomyocytes, glutamate failed to protect cells against H/R injury when NCX1 was inhibited at the reoxygenation (Fig. 2). Interestingly, we found that SN-6 applied at 1 μM only during the reoxygenation phase failed to protect H9c2-NCX1 cells or rat adult cardiomyocytes against H/R injury, in line with previous data obtained in different cell lines expressing NCX1^34^.

In the heart, glutamate is one of the main constituent of the free intracellular amino acid pool^7^. Beyond its role as building block during anabolic macromolecular synthesis, glutamate acts as a key metabolite of myocardial energy metabolism. Indeed, its activity in coupling cytosolic and intra-mitochondrial energetic states through the malate-aspartate shuttle is well known^9^. Some experimental interventions aimed to improve cardiac metabolism against I/R challenge are based on the attempt to favor glutamate or glutamine utilization, through the classical ischemic preconditioning, the increase in glutamate transporter activity or the perfusion of glutamine supplement^35–37^. The administration of high doses of glutamate during post-ischemic reperfusion has been shown to improve left ventricular function^38^, and, in line with this observation, we found that glutamate supplementation from the start of the reoxygenation phase dramatically improved cell viability in our cardiac models of H/R. Specifically, protection was observed in H9c2-NCX1 cells, but not in H9c2-WT exposed to glutamate. Moreover, in the H9c2-NCX1 cells, the ability of glutamate to ameliorate viability was abolished by the NCX1 inhibitor SN-6. Taken together, these findings provide the first clear evidence for the involvement of NCX1 activity in promoting glutamate-induced cell survival.

Since an important step in the recovery of cardiac cells upon reperfusion is the resumption of the oxidative phosphorylation, we explored the effect of glutamate supplementation on ATP production and the involvement of NCX1 in such metabolic response. As expected, in H9c2-NCX1 cells subjected to H/R challenge, ATP levels were dramatically reduced after already 1 h of reoxygenation. Interestingly, this drop in ATP content was fully counteracted by glutamate administration in the first hour of reoxygenation. In connection with these results, a previous work by Kristiansen and coworkers showed that, in a rat isolated perfused heart model, the administration of exogenous glutamate from the beginning of the reperfusion reduces infarct size to the same extent as its administration during both ischemia and reperfusion, indicating that glutamate main effect is linked to the latter phase^5^. Additionally, we found that the NCX1 inhibitor SN-6 abolished the ability of glutamate to ameliorate ATP production. Our data demonstrated that NCX1 may play a critical role in the glutamate-induced ATP synthesis under both pathological and physiological conditions^17,18^.

It is known that glutamate can get access to the mitochondrial matrix via the aspartate/glutamate carriers, a required component of the malate/aspartate shuttle^39,40^. We have recently proposed an alternative and innovative pathway, whereby EAAC1 - a member of the EAATs family^25,41,42^ - and NCX1 cooperate in order to favor glutamate entry into the cytoplasm and then into the mitochondria, stimulating ATP synthesis^17,18^. Therefore, once confirmed the requirement of a functional NCX1 for glutamate to improve cell survival by enhancing the ATP response under hypoxic conditions, an involvement of EAATs was also tested. The ability of glutamate to stimulate ATP recovery and restore cell viability was fully abolished in the presence of the non-transportable EAATs inhibitor DL-TBOA (300 µM), confirming that glutamate entrance into the cells was mediated by EAATs.

We have previously demonstrated that when H9c2-NCX1 (but not H9c2-WT) are acutely exposed to glutamate an increase of the reverse mode of NCX1 (i.e. Ca^2+^ influx/Na^+^ efflux exchange cycle) is observed, and this increase is selectively inhibited by NCX or EAATs blockers^17^. Such increase of the reverse mode of NCX1 develops within seconds and relies on the presence of extracellular glutamate that, being cotransported with Na^+^ into the cell via EAATs, influences both Na^+^ gradient across plasma membrane and membrane potential^17^. Overall, results from our and other groups^43,44^ lend support to the existence of a functional coupling between EAAT and NCX transporters in different cell types, whereby the EAAT-induced NCX reverse mode maintains the Na^+^-driving force for an effective glutamate uptake. After being picked up by cells, glutamate can be used as metabolic fuel for mitochondrial ATP synthesis, and in this process, Ca^2+^ signals originated by the reversed NCX activity can also play a role^17^.

The glutamate-induced recovery in ATP-linked respiration and other OCR parameters indicates an improved activity of ETC, which otherwise would be unable to support the increased ATP demand (and thereby viability) during reoxygenation phase. Overall, our data indicate that H/R H9c2-NCX1 cells treated with glutamate are better equipped to function in conditions of increased energy needs. The improvement of oxygen consumption by glutamate in H/R was significantly suppressed when NCX1 was blocked with SN-6, further highlighting the key role of this exchanger in glutamate-dependent protection of ischemic myocytes.

The mechanisms underlying the glutamate-induced decrease of ECAR in H/R cells were no further investigated in the present study. Albeit speculative, several mechanisms may come into play. It has been shown that in ischemic cardiomyocytes glutamate decreases lactate levels by shunting pyruvate to alanine^45–48^, and promotes diversion of glucose into glycogen rather than undergoing glycolytic oxidation^38^. In any case, the decrease of glycolytic rate induced by glutamate in H/R H9c2-NCX1 cells may also contribute to the observed protection. This is because glycolysis produces significant level of acidic by-products (i.e. lactate), and proton accumulation accounts for a substantial proportion of dysfunctions of myocytes in ischemic settings^1^.

During reoxygenation the molecular oxygen reintroduced to hypoxic myocytes can be converted to oxygen free radicals above viable levels^49^, and glutamate can improve the reducing power of myocytes^4^. Therefore, it is possible that the restoration of energetic metabolism and the preservation of free radical scavengers^6^ acted as synergistic components of glutamate-induced protection. Indeed, we found that H/R injury increased reactive oxygen species (ROS) in H9c2-NCX1 cells and that glutamate partially, but significantly, reduced such increase (Supplementary Fig. 3).

Our use of glutamate at 1 mM, which is somehow higher than basal plasma levels (between 0.05-0.2 mM, depending on mammalian species^6,38^), was based on the following considerations. First, at the concentration tested in this study, glutamate has no evident toxic effect on H9c2 cells and rat adult cardiomyocytes. Second, protection against ischemic injury typically requires glutamate supplementation in the millimolar range^5,6,38,50^. Third, although glutamate has very large muscle/plasma ratios at the baseline, during myocardial ischemia such large concentration gradient dissipates^4^ and high exogenous concentrations of glutamate are required to compensate this loss and enable adequate intracellular glutamate loading^6^. Fourth, ATP synthesis in H9c2-NCX1 cells is significantly stimulated when glutamate is used at 1 mM concentration^17^ (Fig. 4a).

Considering that ionic disturbances occurring during H/R are leading causes of cell death^2^, we investigate whether the ability of NCX1 to control intracellular Ca^2+^ levels might be involved in the glutamate-induced cardioprotection. Experiments performed by using the fluorescent Ca^2+^ indicator Fluo-4 showed an increased NCX1 reverse-mode activity in cells that were subjected to H/R, rather than in controls. It is interesting to note that the increase in NCX1 activity tended toward normalization when cells were reoxygenated in the presence of 1 mM glutamate. We hypothesize that ATP produced from glutamate by H/R cells can support Na^+^/K^+^-pump and Ca^2+^-ATPase to restore intracellular Na^+^and Ca^2+^ levels, so that the NCX-driven Ca^2+^ overload is limited and cell survival promoted. An alternative (but not exclusive) explanation could rely on the finding that increased ATP levels, in the presence of Ca^2+^ transients, may evoke a rapid massive endocytosis, which can involve NCX1^51^. It is possible to speculate that glutamate-enhanced ATP cellular content may serve as a trigger for rapid massive endocytosis, which in turn may remove NCX1 from the cell surface, thereby limiting its activity. Since both the hypothesis depends on the glutamate entry into the cells, the involvement of the EAATs in this metabolic pathway was also confirmed by testing NCX1 activity in the presence of DL-TBOA. As expected, we found that glutamate failed to attenuate the Ca^2+^ increase evoked by H/R insult when cells were incubated with the inhibitor DL-TBOA.

Collectively, our data provide clear evidence that glutamate supplementation from the beginning of the reoxygenation phase can positively affect cell viability by sustaining the oxidative metabolism and increasing ATP content, with NCX1 and EAATs playing a critical role. In particular, as for normoxic conditions, we propose an alternative and regulated mechanism whereby EAATs activity would stimulate NCX1 reverse mode of operation, leading to an increase in mitochondrial Ca^2+^ concentration, to a higher physiological steady-state level likely stimulating Ca^2+^-sensitive dehydrogenase activity and the rate of ATP synthesis. Indeed, Ca^2+^ may play a dual role within the cells: on one hand this ion can be essential to stimulate ATP synthesis, on the other hand it can be harmful, by triggering cell death pathways^52^. There must be a critical point representing the boundary between cytoprotective and cytotoxic effects related to the increase in [Ca^2+^]_i_, and our results demonstrate that this point might also critically depend upon NCX1 activity.

## Methods

### Cell Culture

H9c2 Wilde Type (WT), a clonal cell line derived from embryonic rat heart, were purchased from the American Type Culture Collection (CRL-1446). H9c2-NCX1 cells stably expressing NCX1 were obtained as previously described^17,20^. Both cell lines were cultured as monolayer to sub-confluence in polystyrene dishes (100 mm diameter) and grown in Dulbecco’s Modified Eagle Medium, DMEM (Invitrogen, Carlsbad, CA) supplemented with 10% heat inactivated fetal bovine serum (Invitrogen), 1% L-glutamine (200 mM) (Invitrogen), 1% sodium pyruvate (100 mM) (Invitrogen), 100 IU/ml penicillin (Invitrogen), and 100 μg/ml streptomycin (Invitrogen). Cells were grown in a humidified incubator at 37 °C in a 5% CO_2_ atmosphere.

### Isolation of rat adult ventricular cardiomyocytes

One-month old male Wistar rats (Charles River, Lecco, Italy) were used for cardiomyocytes isolation. The animal protocol was approved by the Ethic Committee for Animal Experiments of the University Politecnica of Marche (Ref no. 721/2015-PR). All the experiments were conducted in strict accordance with the guidelines of the Italian Ministry of Health (D.L.116/92 and D.L.111/94-B). All efforts were made to minimize the number of animals used as well as their suffering.

Cardiomyocytes were isolated by Collagenase type II-CLS2 (Worthington Biochemical Corporation, Lakewood, NJ) digestion using a modified Langendorff perfusion system as previously described^19,20^ (See Supplementary Information for further details).

### *In vitro* hypoxia/reoxygenation challenge

The day before the H/R experiment, cells were plated in 6 multiwell plates (120,000 cells/well for H9C2 cells or 10,000 cells/cm^2^ for cardiomyocytes). Hypoxia was induced in an airtight chamber in which O_2_ was replaced with N_2_ in a glucose-free Tyrode’s solution containing (in mM): NaCl 137, KCl 2.7, MgCl_2_ 1, CaCl_2_ 1.8, NaH_2_PO_4_ 0.2 and NaHCO_3_ 10, pH 7.4. After closing all sealable connectors, the chamber was transferred to an incubator and the cells were subjected to hypoxia (as described in Fig. 1) at 37 °C. Reoxygenation was initiated by opening the chamber and then replacing the glucose-free Tyrode’s solution with fresh Tyrode’s solution containing 5.5 mM glucose^53^. The cells were then maintained in the incubator under an atmosphere of 5% CO_2_, at 37 °C, as described in Fig. 1. Glutamate supplementation does not significantly modifies medium osmolarity.

### Evaluation of cell viability

H/R-induced cell injury was quantified by measurement of lactate dehydrogenase (LDH) activity released from the cytosol of damaged cells in the experimental media^53^, and by the method of double staining with FDA/PI^21^. At the end of the H/R experiment, 100 μl of cell culture medium were removed and added to a 96 well plate. Then, 100 μl of the reaction mixture (Diaphorase/NAD^+^ mixture premixed with iodotetrazolium chloride/sodium lactate) were added to each well and the plate was incubated for 30 min at room temperature, protected from light. LDH activity was assessed by reading the absorbance of the sample medium at 490 nm in a Victor Multilabe Counter plate reader (Perkin Elmer, Waltham, MA, USA). For FDA/PI staining, cells were plated on glass coverslips and subjected to H/R. Afterwards, cells were treated with 36 μM FDA (Sigma) and 7 µM PI (Calbiochem., San Diego, CA, U.S.A.) for 10 min at 37 °C in PBS. Stained cells were examined immediately with an inverted Zeiss Axiovert 200 microscope (Carl Zeiss, Milan, Italy) and then analyzed. When FDA crosses the cell membrane it is hydrolyzed by intracellular esterases producing a green-yellow fluorescence. Cell damage curtails FDA staining and allows cell permeation by PI that, interacting with nuclear DNA, yields a bright red fluorescence^21^.

### Analysis of ATP production

ATP synthesis was evaluated using a commercially available luciferase-luciferin system (ATPlite, Perkin Elmer, Waltham, MA). The day before the experiment, cells were plated (5,000 cells/well) in 96 multiwell plates. The day after, cells were first washed with Tyrode’s solution containing (in mM): NaCl 137, KCl 2.7, MgCl_2_ 1, CaCl_2_ 1.8, NaH_2_PO_4_ 0.2, NaHCO_3_ 10, glucose 5.5 mM, pH 7.4 and then exposed to different glutamate concentrations (0.5 and 1 mM) in the same Tyrode’s solution for 1 h at 37 °C^17^. When ATP content was evaluated after H/R, glutamate and the specific pharmacological tools were added at the beginning of the reoxygenation phase and maintained for 1 h. After the incubation period, ATP levels were analyzed with a luminescence counter (Victor Multilabel Counter, Perkin Elmer) and normalized to the respective protein content^17,18^.

### Bioenergetic analysis

Seahorse XF24 Extracellular Flux Analyzer (Seahorse Bioscience, North Billerica, MA, USA) was used to detect oxygen consumption rate (OCR) and extracellular acidification rate (ECAR), representing oxidative phosphorylation and glycolysis, respectively, as previously described^54,55^. The general scheme of the mitochondrial stress test is shown in Fig. 5a. Oligomycin (1.5 μM), FCCP (2 μM), rotenone/antimycin A (0.5 μM) were sequentially introduced to measure basal respiration, ATP production, proton leak, maximal respiration, spare respiratory capacity, and non-mitochondrial respiration. Maximal respiratory capacity was estimated by inducing maximal OCR via chemical dissipation of the mitochondrial membrane potential with the protonophore FCCP on the background of oligomycin (used to prevent the ATP-consuming reverse activity of ATP synthase, which may lead to cellular metabolic dysfunction and death). Maximal respiratory capacity is a measure of the maximal ability of the ETC to produce energy. Spare respiratory capacity is derived from the difference between maximal OCR and basal respiration. A cell with a larger spare respiratory capacity can produce more ATP to maintain adequate levels of energetic molecules and overcome more stress.

The general scheme of glycolysis stress test is shown in Fig. 5b. Sequential injections of 3 μM rotenone (to block complex I, thereby eliminating mitochondrial respiration and force cells to rely on glycolysis), 10 mM glucose, and 100 mM 2-deoxyglucose (2-DG; glucose analog and inhibitor of glycolytic ATP production) were used to measure glycolysis, glycolytic capacity and allow estimation of glycolytic reserve and non-glycolytic acidification.

H9c2 cells (40,000 cells/well) were seeded on the XFp cell culture mini plates (Seahorse Bioscience, Billerica MA, USA) and subjected to the H/R challenge (Fig. 1). At the end of the first hour of reoxygenation, the Tyrode’s solution was replaced with 500 μl/well of XF24 running media. The plates were pre-incubated at 37 °C for 20 min in the XF Prep Station incubator (Seahorse Bioscience, Billerica MA, USA) in the absence of CO_2_ and then run on the XF24 analyzer to obtain OCR and ECAR.

OCR and ECAR were recorded during specified programmed time periods (three readings each) as the average numbers between the injections of inhibitors mentioned above. The final data calculation was performed after the readings had been normalized for total protein/well.

### Western blotting

Protein extraction and western blotting analysis were performed as previously described^19^. Immunoblots were probed overnight at 4 °C with the appropriate primary antibody: NCX1^17,19^ (R3F1, Swant, Bellinzona, Switzerland), dilution 1:500; mouse anti-EAAC1^17^ (Chemicon International, CA, USA), dilution 1:1,000; rabbit anti-GLAST and rabbit anti-GLT1^17^ (Alpha Diagnostic International) both used at 1:1,000 dilution. β-actin (1:10,000; A5316, Sigma) was used as loading control^19^ (See Supplementary Information for further details).

### Analysis of NCX1 activity

H9c2-NCX1 cells, cultured on 25 mm coverslip, were loaded with 4 µM Fluo-4/AM for 30 min in the dark at room temperature (Molecular Probe, Eugene, OR), in a standard solution containing (in mM): NaCl 140, KCl 5, MgCl_2_ 1, CaCl_2_ 2, glucose 10, HEPES 20, pH 7.4 adjusted with NaOH. At the end of the Fluo-4/AM loading period, cells were washed and left in the standard solution for further 10 min to allow the complete de-esterification of the dye. Then the coverslips were placed into a perfusion chamber mounted onto the stage of an inverted Zeiss Axiovert 200 microscope. NCX1 activity was evaluated as Ca^2+^ uptake through the reverse mode by switching the standard solution to a Na^+^-free solution containing (in mM): LiCl 140, KCl 5, MgCl_2_ 1, CaCl_2_ 2, glucose 10, HEPES 20, pH 7.4 adjusted with LiOH. [Ca^2+^]_i_ was measured by single-cell computer-assisted videoimaging using a LSM 510 confocal system (Carl Zeiss). Cells were treated according to the protocol schemes reported in Fig. 1. In particular, cells were exposed to glutamate and/or transporter inhibitors only during the reoxygenation phase or, for sham-treated cells (not subjected to hypoxia), under normoxia for equivalent length of time, and were not included in solutions used for Fluo-4/AM loading or fluorescence monitoring. Excitation light was provided by an argon laser at 488 nm and the emission was time-lapse recorded at 505-530 nm. Images were acquired every 5 s. Analysis of fluorescence intensity was performed off-line after images acquisition, as described before^17,20^.

### Drug and chemicals

SN-6 and DL-TBOA were obtained from Tocris. All the other chemicals were of analytical grade and were purchased from Sigma.

### Statistical analysis

Data were expressed as mean ± S.E.M. Values less than 0.05 were considered to be significant. Differences among means were assessed by Student’s t-test or one-way ANOVA followed by Dunnet’s *post hoc* test. Statistical comparisons were carried out using the GraphPad Prism 5 software (GraphPad Software Inc., San Diego, CA).

## Electronic supplementary material

Supplementary Information
