# Supplementary material for: Essential role of the Na+-Ca2+ exchanger (NCX) in glutamate-enhanced cell survival in cardiac cells exposed to hypoxia/reoxygenation
Source: Sci Rep. 2017 Oct 12;7:13073. doi: 10.1038/s41598-017-13478-x (PMC5638850; doi:10.1038/s41598-017-13478-x)
Supplement: Supplementary file 1 — Supplementary Information [file 41598_2017_13478_MOESM1_ESM.pdf]

Essential role of the Na$^+$-Ca^{2+}exchanger (NCX) in glutamate-enhanced cell survival in cardiac cells exposed to hypoxia/reoxygenation

Marta Maiolino§, Pasqualina Castaldo§, Vincenzo Lariccia*, Silvia Piccirillo, Salvatore Amoroso and Simona Magi

Department of Biomedical Sciences and Public Health, School of Medicine, University "Politecnica delle Marche", Via Tronto 10/A, 60126, Ancona, Italy

§These authors equally contributed to this paper

*Correspondence and requests for materials should be addressed to

Vincenzo Lariccia, PhD

Department of Biomedical Sciences and Public Health, School of Medicine

University "Politecnica delle Marche", Via Tronto 10/A, 60126, Ancona, Italy

Phone: +39 071 2206037; Fax:+39 071 2206040

E-mail: v.lariccia@univpm.it

Supplementary information

Materials and methods

Isolation of rat adult ventricular cardiomyocytes

One-month old male Wistar rats (Charles River, Lecco, Italy) were used for cardiomyocytes isolation. The animal protocol was approved by the Ethic Committee for Animal Experiments of the University Politecnica of Marche (Ref no. 721/2015-PR). All the experiments were conducted in strict accordance with the guidelines of the Italian Ministry of Health (D.L.116/92 and D.L.111/94-B). All efforts were made to minimize the number of animals used as well as their suffering. Cardiomyocytes were isolated by Collagenase type II-CLS2 (Worthington Biochemical Corporation, Lakewood, NJ) digestion using a modified Langendorff perfusion system as previously described[1,2]. Briefly, rats were anesthetized with 4% isoflurane in 100% O_2 and then intraperitoneally injected with 1 ml of heparin (5000 IU/ml). After 10 min, the chest was opened and 1 ml of heparin (160 UI/ml) was injected into the right atrium. Then the heart was quickly excised, attached to the modified Langendorff perfusion system and retrogradely perfused with an O_2-saturated HEPES buffered solution containing (in mM): NaCl 140, KCl 4, HEPES 10, Na_2HPO4 0.5, $MgCl_2$ 1, $CaCl_2$ 1.5, glucose 15, pH 7.4 adjusted with NaOH. After 2 min, the solution was switched to nominally Ca^{2+}-free HEPES buffered solution for 5 min, followed by perfusion with the same solution containing 100 μM EGTA for 2 min. After that, the heart was perfused with enzyme solution (150-200 U/ml) containing 30 µM blebbistatin (Sigma, Milan, Italy) for about 10 min. The digestion was stopped by perfusing HEPES buffered solution containing 10% FBS. The heart was then deprived of atria and aorta and put on a sterile petri dish containing the perfusion solution without Ca^{2+}, with 100 μM EGTA and 30 μM blebbistatin. Single cells were isolated by mechanical dispersion and harvested after filtration through a nylon mesh. Ca^{2+} was then gradually reintroduced[1,3]. Once the cardiomyocytes were Ca^{2+}-tolerant, they were plated at a density of

10,000 cells/cm^2 on laminin coated dishes, and cultured in M-199 medium containing L-glutamine, $NaHCO_3$ and Earle's salts, supplemented with 0.2% bovine serum albumin, 1X insulin-transferrin-selenium (Gibco, Grand island, NY, USA), 2 mM L-carnitine, 5 mM creatine, 3 mM taurine, 1% penicillin-streptomycin (Invitrogen), at 37°C in 5% CO2 atmosphere[3].

Western blotting

Protein extraction and western blotting analysis were performed as previously described[1]. Cells were lysed using a protein lysis buffer containing (in mM): NaCl, 150; Tris-HCl (pH 7.4), 10; EDTA (pH 8.0), 1; SDS 1%, and a protease inhibitor cocktail mixture (Roche Diagnostics).

Equal protein amounts (50 μg, determined by Bradford method) were subjected to SDS-PAGE and transferred to polyvinylidine difluoride (PVDF) membranes (Immobilon Transfer Membranes, Millipore Co., Bedford, MA, USA). Immunoblots were probed with the appropriate primary antibody overnight at 4°C, followed by horseradish peroxidase-conjugated secondary antibody. An enhanced chemiluminescence detection system (Super Signal West Femto kit, Thermo Scientific, Milano, Italy) was used to detect bound antibodies. Images were captured and stored on a ChemiDoc station (BioRad, Milan, Italy). Band densities were analyzed with the Quantity One (Bio-Rad) analysis software and normalized to β-actin.

Antibodies

NCX1 protein was detected by using a commercially available mouse monoclonal IgG antibody[1,4] (R3F1, Swant, Bellinzona, Switzerland, dilution 1:500). The following primary antibodies were used to detect EAATs: mouse anti-EAAC1[4] (Chemicon International, CA, USA, dilution 1:1000), rabbit anti-GLAST and rabbit anti-GLT1[4] (both purchased from Alpha Diagnostic International and used at 1:1000 dilution). β-actin (1:10000; A5316, Sigma) was used as loading control[1].

ROS detection

ROS production was assessed by using the cell-permeative probe 2′7′-dichlorodihydrofluorescein diacetate[5] (H_2DCFDA, Calbiochem, Vimodrone, Italy). Upon entry into the cytoplasm, this probe is hydrolyzed to a non-fluorescent polar derivative (H_2DCF) by cellular esterases[6]. H_2DCF is membrane-impermeable and rapidly oxidized by intracellular ROS to a highly fluorescent compound, 2′7′-dichlorofluorescein (DCF). Briefly, H9c2 cells were plated in 12 multiwell plates. After H/R challenge, cells were incubated in the dark for 30 min with 20 μM H_2DCFDA. Cells were subsequently washed twice with PBS and DCF fluorescence was measured using a multilabel microplate reader (Victor Multilabel Counter, Perkin Elmer) at excitation and emission wavelengths of 485 and 535 nm, respectively.

Figure legends

Figure S1. Effect of H/R injury on cell survival in H9c2 cells. (**a**) Extracellular LDH activity measured 5 h after the hypoxic insult (3 h) both in H9c2-WT and in H9c2-NCX1 cells. Differences among means were assessed by one-way ANOVA followed by Dunnet's *post hoc* test. Each column represents the mean ± S.E.M. of almost 6 independent experiments performed in duplicate. *p<0.001 versus any other group; #p<0.001 versus H/R-WT group. (**b**) Evaluation of H9c2 survival by PI/F assay showing the intravital staining that yields green-yellow fluorescence (FDA) for vital cells, and red fluorescence (PI) for dead cells, under normoxic (CTL) and H/R conditions. Red death cells tend to detach, thus images could underestimate their exact number. Images are representative of 3 independent experiments.

CTL= control; H/R= hypoxia/reoxygenation.

Figure S2. Effect on mitochondrial function of glutamate-induced ATP synthesis in H9c2-NCX1 cells subjected to H/R. Intracellular ATP content evaluated after H/R in the presence of 1 mM glutamate, alone or in combination with 3 µg/ml olygomicin, added at the beginning of the reoxygenation phase and maintained for 1 h. ATP levels were normalized to the respective sample protein content. Differences among means were assessed by one-way ANOVA followed by Dunnet's *post hoc* test. Each column represents the mean ± S.E.M. of 6 independent experiments performed in triplicate. *p<0.001 versus and H/R+G, #p< 0.001 versus H/R and versus H/R+OLIG and p<0.01 versus H/R+G+OLIG, §p<0.001 versus CTL and H/R+G.

CTL= control; H/R= hypoxia/reoxygenation; G= glutamate; OLIG= oligomycin.

Figure S3. Effect of glutamate exposure on ROS production in H9c2-NCX1 cells subjected to H/R. Intracellular ROS production assessed after H/R challenge by evaluation of the DCF fluorescence in the presence of 1 mM glutamate during the reoxygenation phase. DCF fluorescence values were normalized to untreated control and expressed as percentage. Differences among means

were assessed by one-way ANOVA followed by Dunnet's *post hoc* test. Each column represents the mean ± S.E.M. of almost 6 independent experiments performed in triplicate. *$p<0.001$ versus CTL and $p<0.05$ versus H/R+G.

CTL= control; H/R= hypoxia/reoxygenation; G= glutamate.

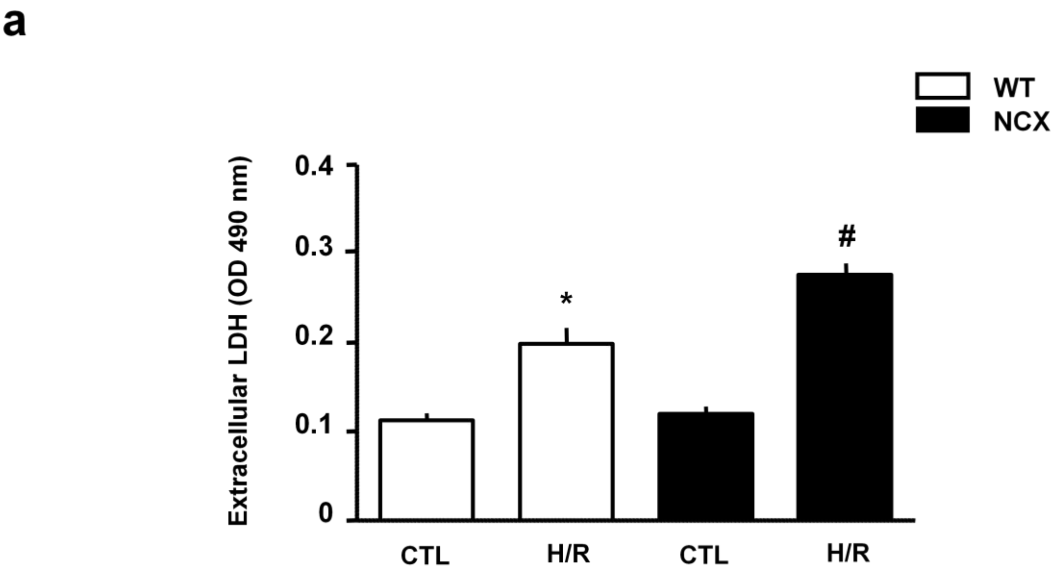

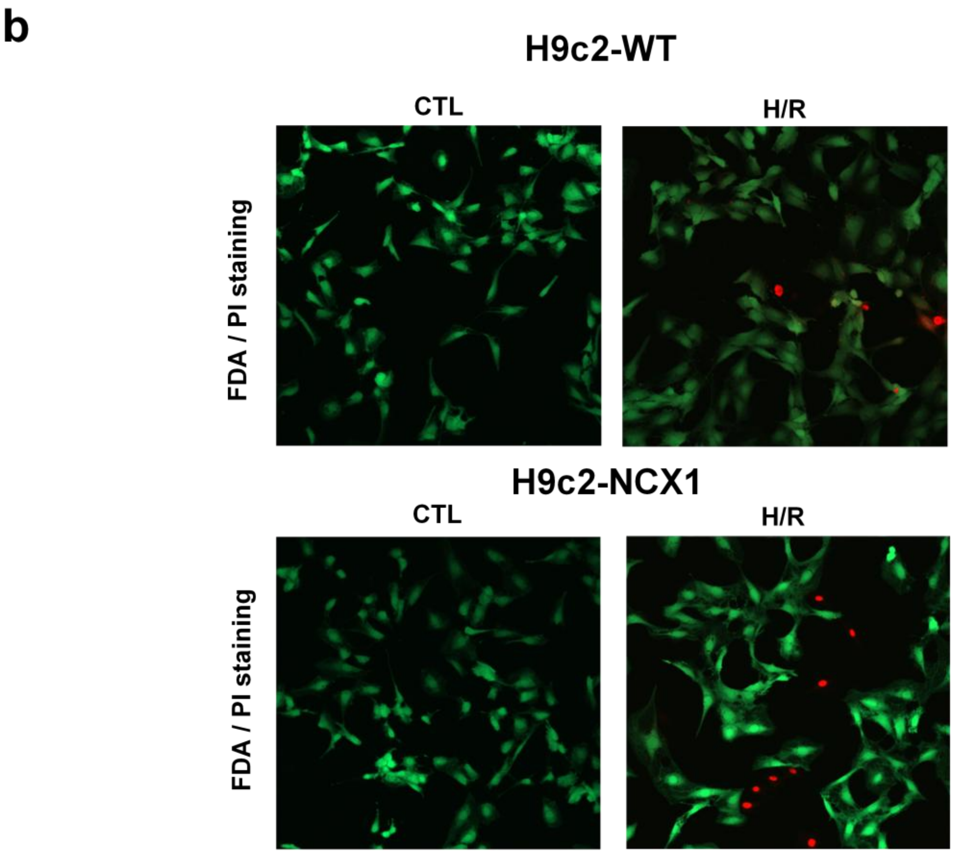

Maiolino et al Figure S1

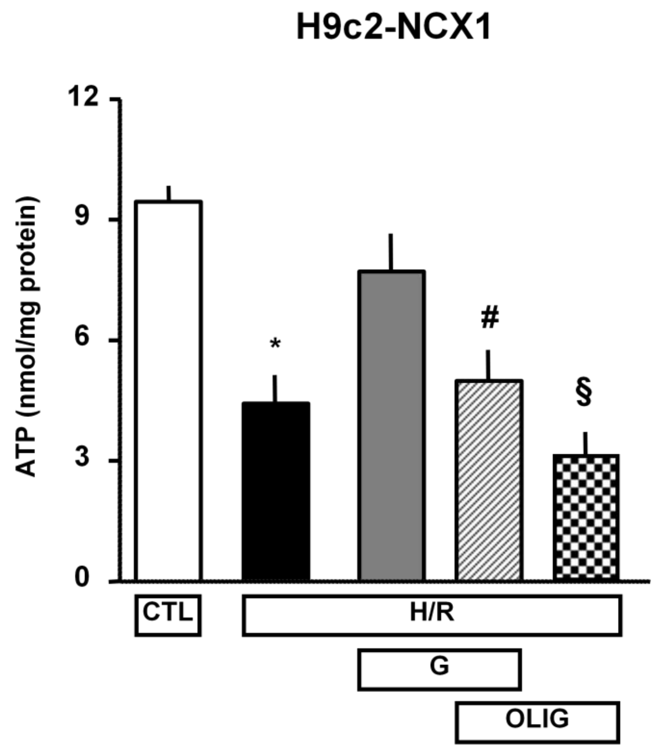

Maiolino et al Figure S2

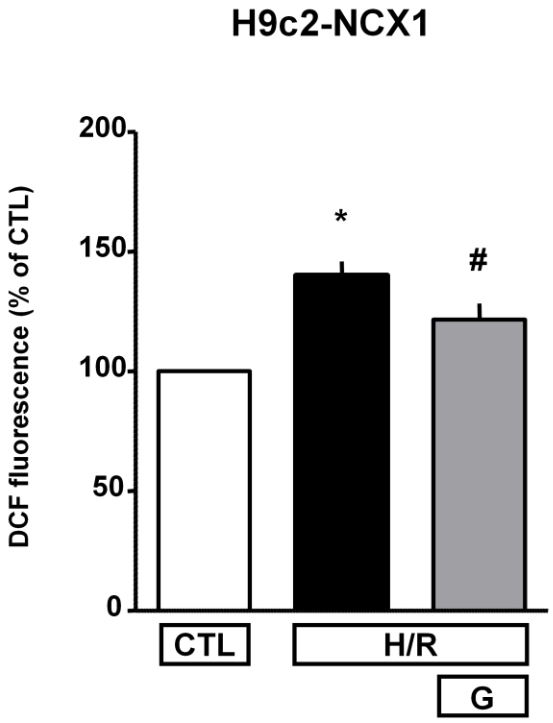

Maiolino et al Figure S3

Figure 7

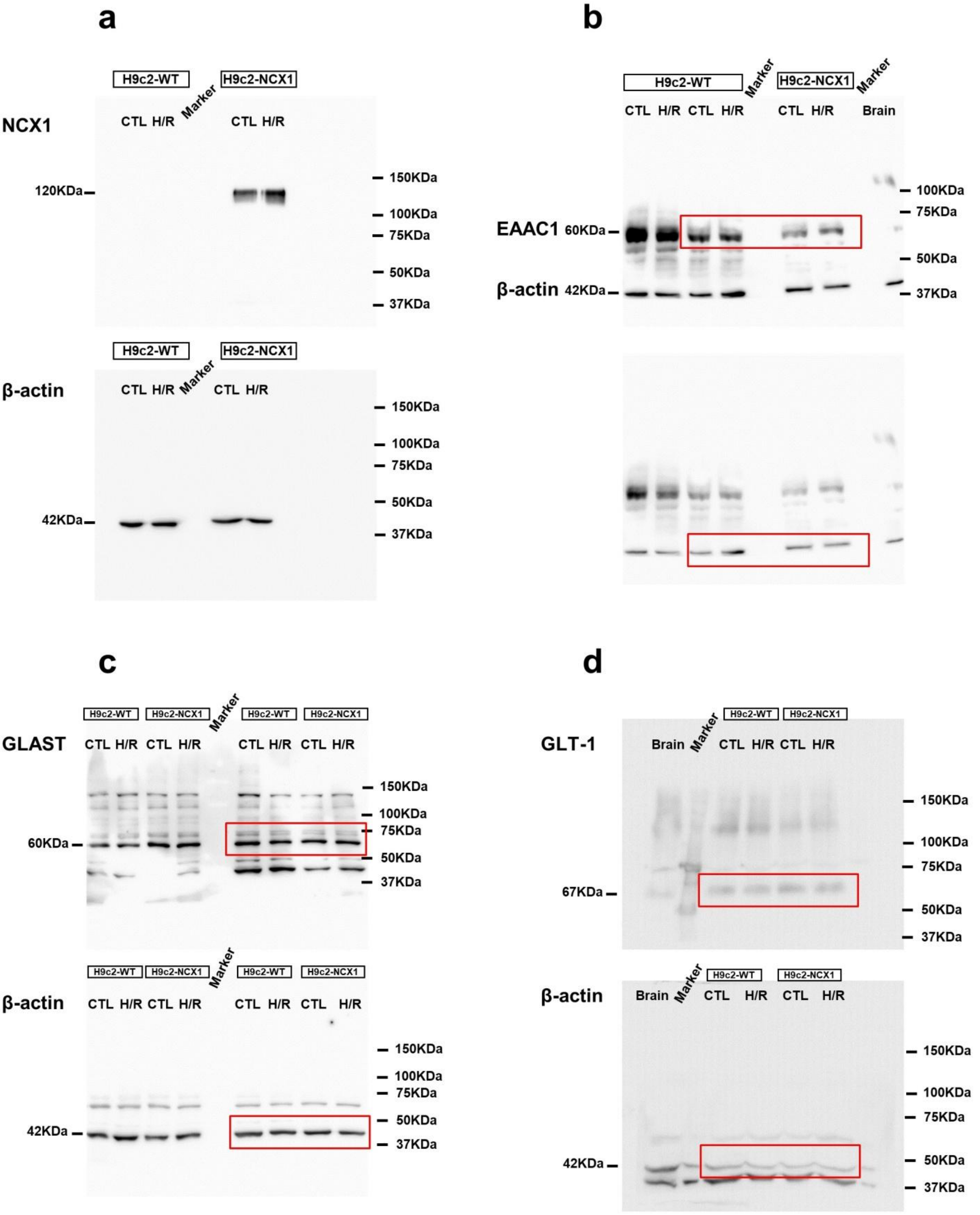

Maiolino et al Figure S4

References

1 Castaldo, P. *et al.* Na+/Ca2+ exchanger 1 inhibition abolishes ischemic tolerance induced by ischemic preconditioning in different cardiac models. *Eur J Pharmacol* **794**, 246-256 (2016).

2 Magi, S. *et al.* Gram-negative endotoxin lipopolysaccharide induces cardiac hypertrophy: detrimental role of Na(+)-Ca(2+) exchanger. *Eur J Pharmacol* **746**, 31-40 (2015).

3 Guaiquil, V. H., Golde, D. W., Beckles, D. L., Mascareno, E. J. & Siddiqui, M. A. Vitamin C inhibits hypoxia-induced damage and apoptotic signaling pathways in cardiomyocytes and ischemic hearts. *Free Radic Biol Med* **37**, 1419-1429 (2004).

4 Magi, S. *et al.* Glutamate-induced ATP synthesis: relationship between plasma membrane Na+/Ca2+ exchanger and excitatory amino acid transporters in brain and heart cell models. *Mol Pharmacol* **84**, 603-614 (2013).

5 Amoroso, S., D'Alessio, A., Sirabella, R., Di Renzo, G. & Annunziato, L. Ca(2+)-independent caspase-3 but not Ca(2+)-dependent caspase-2 activation induced by oxidative stress leads to SH-SY5Y human neuroblastoma cell apoptosis. *J Neurosci Res* **68**, 454-462, doi:10.1002/jnr.10199 (2002).

6 Wang, H. & Joseph, J. A. Quantifying cellular oxidative stress by dichlorofluorescein assay using microplate reader. *Free Radic Biol Med* **27**, 612-616 (1999).
